# Supplementary material for: Improving traffic accident severity prediction using MobileNet transfer learning model and SHAP XAI technique
Source: PLoS One. 2024 Apr 9;19(4):e0300640. doi: 10.1371/journal.pone.0300640 (PMC11003624; doi:10.1371/journal.pone.0300640)
Supplement: Attachment — Submitted filename: Reply Letter__PONE_D_23_34929.pdf [file pone.0300640.s001.pdf]

Manuscript ID: PONE-D-23-34929

Article title: Improving Traffic Accident Severity Prediction Using MobileNet Transfer Learning Model and SHAP XAI Technique

January 20, 2024

Dear Academic Editor,

First of all, we would like to extend our sincere gratitude to Editor in Chief, Academic Editor, for allowing a re-submission of our manuscript, with an opportunity to address the reviewer's comments. We greatly appreciate the Reviewers' time spent reviewing the paper and assisting in its improvement. These comments are very helpful for revising and improving the paper, as well as the important guiding significance to the other research. We have studied the comments very carefully and made the corrections that we hope to meet with the final approval. We have done our best to modify the paper to reflect all the comments made by anonymous reviewers. These changes are fully addressed in this reply letter.

Below, we provide point-to-point responses explaining how we have addressed each of the Reviewer's comments. For clarity, our responses have been written in blue color. whenever we copy text from the manuscript, it is given in red color.

REVIEWER 1

Concern 1

The use of precise technical language: Avoid use of first person pronouns (I, we, us, etc.). Use commonly used technical terms, such as "lack" instead of "dearth".

Response: We are thankful for your insightful comment. Respected reviewer, We have corrected it.

Concern 2

Reference number [3] is not "recent", furthermore, it is not properly written in the reference list.

Response: The authors are grateful for your valuable suggestions. We have updated the Reference number [3].

Concern 3

The full form of PART is missing.

Response: We are thankful for your insightful comment. Respected reviewer, We have provided the full form of PART which is "Partial Decision Trees" in the paper.

Concern 4

The dataset is heavily biased towards non-fatal crashes. In that case, some balancing technique must be

applied to have a valid model. IF not, then the model accuracy must be shown for each prediction class to show that the model is valid even without the use of balancing.

Response: Respected reviewer, we have extracted 2500 records from each class using the stratified parameter. I also provided the class-wise accuracy of each model in the tabular form in the paper.

Table 1: Class-wise results of transfer learning models for traffic accident severity detection.

**Models**	**Fatal**	**Serious Injury**	**Minor Injury**	**Non-Injury**
ResNET	88	96	97	97
EfficientNetB4	87	94	94	95
InceptionV3	86	93	92	93
Xception	87	94	92	92
MobileNet	94	98	99	99

Concern 5

I believe SHAP analysis could be used for determining feature importance for any of the models. It has been done in the past. So, the only advantage of MobileNet, that could be claimed, is better accuracy. In terms of inherent explanatory power, models like CART are better.

Response: Respected reviewer, The study investigates accident severity prediction using various advanced models, including MobileNet, achieving high accuracy. While acknowledging the interpretability advantage of simpler models like CART, the study balances accuracy and interpretability. It utilizes SHAP values to reveal influential features, enhancing understanding of accident factors. The focus lies in reconciling high accuracy with model transparency to better prevent accidents by comprehensively grasping their contributing factors.

Concern 6

The feature importance found through SHAP should be compared and discussed with other studies from literature.

Response: Respected reviewer, We have updated the section namely "comparison with state-of-the-art". The other studies used in comparison have applied explainable artificial intelligence (XAI) techniques as mentioned in the paper.

A study [2] applied LRP (layer-wise relevance propagation). While LRP is a widely used technique, it has limitations such as sensitivity to hyperparameters and the choice of rules, and its explanations might lack the consistency provided by Shapley values. LRP primarily focuses on local explanations, attributing relevance to features for a specific input instance. The interpretation of LRP results can be more complex, and the choice of specific rules in LRP can impact the explanations.

The other study [1] uses Shapely Decision Plot: The use of the Shapely decision plot technique is commendable, but it is essential to note that different Shapley-based methods can vary in their interpretability and robustness. Shapely Decision Plots are sensitive to the sampling of instances used to compute Shapley values. If the sampled instances do not adequately represent the diversity of the dataset, the decision plot may not accurately reflect the true distribution of Shapley values. Additionally, the reliance on the NHTSA-USA dataset, as outlined, introduces various limitations, including underreporting, inconsistent reporting, and other biases that may impact the reliability of the extracted features.

The proposed approach leverages transfer learning models on a US traffic dataset, enhancing generalizability by utilizing pre-trained models for improved performance and robustness. The introduction of the Shapely Beeswarm plot, based on Shapley values and cooperative game theory, is a noteworthy innovation, providing a theoretically sound and visually interpretable framework for explaining feature contributions

across different predictions. Shapley values contribute to high interpretability and versatility, facilitating a comprehensive understanding of complex models and insights into the decision-making process. The demonstrated robustness, with an accuracy of 98.17%, indicates not only accurate predictions but also meaningful insights into the influential factors driving those predictions.

Concern 7

Disucsion should be extended to show the policy and safety implications of feature importance analysis.

Response: We appreciate your comments. Respected reviewer, We have updated the discussion section.

Concern 8

Why only two studies are selected for comparision of accuracy? Table 1 has a wider range of studies which could also be used for this purpose.

Response: We are thankful for your insightful comment. Respected reviewer, We have selected the most recent studies with the highest results.

Concern 9

Figure 1 shows that data cleaning involved eliminating missing values, which is not mentioned in the text. How the missing values were eliminated? Did it involve eliminating the entire data record missing values?

Response: Respected Reviewer, in this dataset I have a lot of records, So, I prefer deleting entire values for dataset cleaning.

Concern 10

Figure 2 needs a legend.

Response: Respected reviewer, We have updated Figure 2. The updated figure has x-axis and y-axis. Further details of SHAP explanation is added in the paper.

Concern 11

Importance features should be reiterated in the conclusion section.

Response: The authors highly appreciate your valuable suggestions. Respected reviewer, We have updated the conclusion section.

REVIEWER 2

Concern 1

What is the contribution of this study, explain it

Response: We are thankful for your insightful comment. Respected reviewer, This study significantly advances accident severity prediction by introducing an innovative MobileNet model based on transfer learning, showcasing exceptional accuracy. Through comprehensive experimentation involving various machine learning models and explainable techniques like SHAP, it illuminates influential factors driving accident severity. The research's contributions extend to demonstrating model generalizability, affirming its potential beyond the initial dataset. Overall, this study enriches understanding and application in accident

severity prediction, aiding more effective interventions in road safety.

Concern 2

The whole paper is not justified by Grammarly.

Response: The authors are grateful for your valuable suggestions. Respected reviewer, WE have improved the whole manuscript.

Concern 3

Author should cite the related references of traffic prediction such as Bharti, Redhu, P. and Kumar, K., 2023. Short-term traffic flow prediction based on optimized deep learning neural network: PSO-Bi-LSTM. Physica A: Statistical Mechanics and its Applications, 625, p.129001. Yadav, Sunita, and Poonam Redhu. "Driver's attention effect in car-following model with passing under V2V environment." Nonlinear Dynamics (2023): 1-17.

Response: Respected reviewer, We have cited the above-mentioned papers.

Concern 4

The quality of the figures is very low.

Response: Respected reviewer, I have updated all figures quality in the revised version of the paper.

Concern 5

Give more details about MobileNet

Response: Respected reviewer, We have added more details about MobileNet.

Concern 6

What is Shapley values.

Response: Respected reviewer, Shapley values, originating from game theory, offer a fair method to attribute the impact of individual features on a model's predictions. They assess each feature's contribution by considering its interactions with other features, providing insights into their importance in influencing the model's outputs and enhancing the model's interpretability.

Concern 7

Check Ref. [3]

Response: We are thankful for your insightful comment. Respected reviewer , We have updated Ref. [3]

References

[1] Elif Cicek, Murat Akin, Furkan Uysal, and ReyhanMerve Topcu Aytas. Comparison of traffic accident injury severity prediction models with explainable machine learning. *Transportation Letters*, pages 1–12, 2023.

[2] Zekun Yang, Wenping Zhang, and Juan Feng. Predicting multiple types of traffic accident severity with explanations: A multi-task deep learning framework. *Safety science*, 146:105522, 2022.
